# Bone Turnover Markers, DXA and Mineral Metabolism in Long-Term Kidney Transplant Recipients: A Cross-Sectional Comparative Study

**DOI:** 10.3390/jcm15155974

**Published:** 2026-07-31

**Authors:** Nada Akad, Stefana Catalina Bilha, Gianina Dodi, Ioana-Madalina Bilha, Dumitru Branisteanu, Alexandru Florescu, Fawzy Akad, Mihai Marian Hogas, Luminita Voroneanu, Simona Hogas, Cristina Preda, Maria-Christina Ungureanu, Adrian Covic

**Affiliations:** 1 Grigore T. Popa University of Medicine and Pharmacy Iasi, 16 University Street, 700115 Iasi, Romania; 2 Department of Medicine, Charles E. Smith College of Medicine, Florida Atlantic University, Boca Raton, FL 33431, USA

**Keywords:** kidney transplantation, bone turnover markers, bone-specific alkaline phosphatase, chronic kidney disease–mineral and bone disorder, bone mineral density, dual-energy X-ray absorptiometry, skeletal remodeling

## Abstract

**Background:** Bone disease remains a major long-term complication after kidney transplantation. However, the relationship between circulating bone turnover markers (BTMs) and densitometric skeletal parameters in kidney transplant recipients (KTRs) remains incompletely understood. **Methods:** This single-center, cross-sectional comparative study included 50 stable adult KTRs and 56 non-transplanted individuals with dual-energy X-ray absorptiometry (DXA)-confirmed osteopenia or osteoporosis. Serum bone formation markers, bone-specific alkaline phosphatase (BALP) and procollagen type I N-terminal propeptide (P1NP), and bone resorption markers, β-C-terminal telopeptide of type I collagen (β-CTX) and tartrate-resistant acid phosphatase 5b (TRAP5b), were quantified by enzyme-linked immunosorbent assay (ELISA). Bone mineral density (BMD), Z-scores, and Fracture Risk Assessment Tool (FRAX) estimates were obtained by DXA. Between-group comparisons were adjusted for age, sex, body mass index (BMI) and postmenopausal status, with sensitivity analyses restricted to participants with low bone mass. An exploratory receiver operating characteristic (ROC) analysis was performed to assess the association of BTMs with KTR status. **Results**: KTRs showed lower DXA-derived Z-scores at the total hip, femoral neck and lumbar spine despite being younger. After adjustment for age, sex, BMI and postmenopausal status, KTRs had significantly higher BALP (adjusted Δ = 129.96 ng/mL, *p* < 0.001) and PTH, and lower eGFR and magnesium than the reference group, whereas unadjusted differences in P1NP and lumbar spine BMD did not persist; β-CTX and TRAP5b did not differ between groups. BALP remained significantly higher in KTRs across the low-bone-mass restricted and propensity-score overlap analyses. BTMs showed no significant correlations with DXA parameters in KTRs. In the exploratory ROC analysis of study-group status, BALP showed the strongest separation between KTRs and the reference group (AUC = 0.808, *p* < 0.001). **Conclusions:** Long-term KTRs exhibit a distinct bone remodeling profile characterized most consistently by elevated BALP, which remained significant after adjustment and showed the strongest separation regarding KTR status. The limited BTM–DXA associations require confirmation in longitudinal studies.

## 1. Introduction

Kidney transplantation is the preferred treatment for end-stage chronic kidney disease (CKD) and is associated with substantial improvements in patient survival and quality of life. Nevertheless, disturbances in mineral metabolism and skeletal remodeling often remain despite restoration of kidney function. Pre-existing CKD-mineral and bone disorder (CKD-MBD), ongoing disturbances in mineral homeostasis, immunosuppressive therapy, and varying degrees of graft dysfunction may all contribute to skeletal abnormalities after transplantation. Bone metabolism undergoes pronounced alterations during the early post-transplant period, whereas later changes are influenced by graft function and persistent CKD-related mechanisms [1,2].

Consequently, skeletal abnormalities remain common among kidney transplant recipients (KTRs) and represent an important source of long-term morbidity. Despite increasing recognition of post-transplant bone disease, the relationship between bone turnover abnormalities, biochemical markers, and skeletal status remains incompletely understood [3,4].

Current clinical practice guidelines recommend dual-energy X-ray absorptiometry (DXA) as a fundamental component of skeletal assessment in KTRs [2,5]. DXA provides a quantitative assessment of bone mineral density (BMD) and remains the most widely used method for evaluating skeletal status. However, densitometric measurements primarily reflect bone mass at specific anatomical sites and provide limited information regarding the dynamic processes of skeletal remodeling [6].

Bone turnover markers (BTMs) are biochemical indicators of bone formation and resorption that reflect ongoing whole-skeleton metabolic activity and may provide information complementary to DXA [7,8]. Bone-specific alkaline phosphatase (BALP) reflects osteoblastic activity and matrix mineralization, procollagen type I N-terminal propeptide (P1NP) is indicative of type I collagen synthesis, tartrate-resistant acid phosphatase 5b (TRAP5b) reflects the osteoclast number, whereas β-C-terminal telopeptide of type I collagen (β-CTX) reflects osteoclast-mediated degradation of type I collagen [9,10].

BALP and TRAP5b are minimally influenced by renal clearance, making them particularly suitable for use in patients with CKD and kidney transplantation [10,11]. Previous studies have also demonstrated the value of BALP and P1NP in evaluating bone turnover abnormalities associated with CKD, highlighting their potential as non-invasive indicators of skeletal remodeling [12,13,14]. However, the relationship between biochemical markers of bone turnover and DXA-derived skeletal parameters remains heterogeneous and insufficiently characterized in KTRs [15,16,17].

The present study aimed to compare circulating concentrations of BALP, P1NP, β-CTX, and TRAP5b between KTRs and non-CKD reference subjects, evaluate their associations with DXA-derived skeletal parameters, and determine their potential contribution to the characterization of post-transplant bone disease.

## 2. Materials and Methods

### 2.1. Study Design and Objectives

This was an observational, comparative, cross-sectional study conducted between July 2025 and June 2026 that compared KTRs with a non-CKD reference group, both of which underwent biochemical assessment of BTM and DXA evaluation. The primary objective was to determine whether KTRs show a distinct bone remodeling marker profile, defined by BALP, P1NP, β-CTX and TRAP5b, compared with non-CKD subjects who had DXA-documented low bone mass. The secondary objective was to evaluate the correlations between BTMs and calcium–phosphate metabolism parameters in the two groups.

### 2.2. Study Population

Eligible KTR participants were adults (≥18 years and up to 70 years) with a functioning kidney graft and stable graft function at the time of evaluation, who were able to provide written informed consent and complete all study procedures. Of the 345 KTRs initially screened, the final cohort comprised 50 KTRs with a stable kidney graft, defined as the absence of graft loss, acute rejection episodes, acute severe illness, active infection, or clinically significant deterioration of renal function during the preceding 12 months. Patients were excluded in the presence of recent acute graft rejection, return to dialysis, diabetes mellitus, active malignancy, acute infection or other severe illness, liver cirrhosis, recent major fracture, non-renal solid organ transplantation, or active osteoporosis treatment, including bisphosphonates, denosumab, or bone-anabolic agents, other than vitamin D or calcium supplementation, as well as other conditions known to substantially affect bone metabolism.

**Figure 1:**
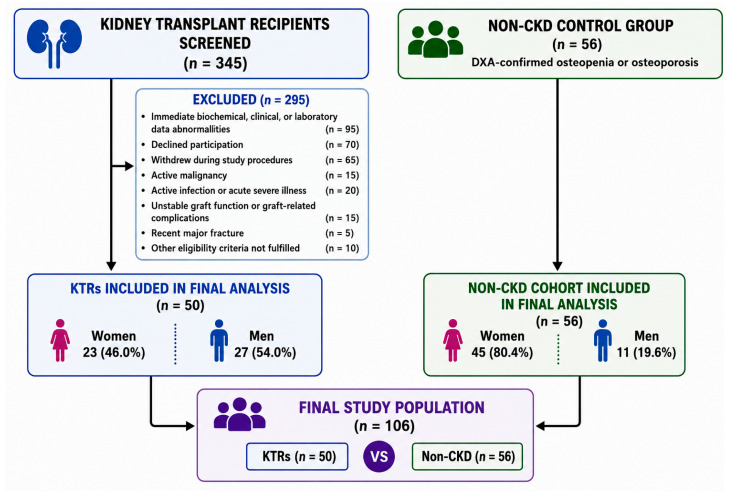
Flow diagram of participant selection and study population composition: KTRs = kidney transplant recipient(s), CKD = chronic kidney disease.

The reference group comprised 56 non-CKD postmenopausal women and men aged >50 years and up to 70 years, with preserved kidney function and DXA-confirmed osteopenia or osteoporosis, who were evaluated in the Department of Endocrinology during the same period and were able to provide informed consent. Exclusion criteria comprised diabetes mellitus, chronic corticosteroid therapy, active malignancy, acute severe illness or infection, liver cirrhosis, recent major fractures, active treatment for osteoporosis, including bisphosphonates, denosumab, or bone-anabolic agents, other than vitamin D or calcium supplementation, or other secondary causes of osteoporosis or conditions known to significantly interfere with bone metabolism. The non-CKD reference group was selected among individuals with DXA-confirmed osteopenia or osteoporosis (according to ISCD criteria [18]) to explore whether KTRs exhibit a distinct BTM profile compared with non-CKD individuals who already have established skeletal involvement, as any differences in bone turnover markers observed in KTRs are less likely to be explained solely by the presence of low bone mass. The participant selection process is summarized in Figure 1.

Given the exploratory nature of the study and the limited number of patients fulfilling all eligibility criteria, no formal a priori sample size calculation was performed. The final sample size was determined by the number of eligible participants who completed the clinical, biochemical, and densitometric assessments during the study period.

### 2.3. Data Collection

Demographic (age, sex), anthropometric (body mass index (BMI)), clinical (smoking status, menopausal status, previous fragility fractures), biochemical (serum calcium, phosphorus, magnesium, albumin, parathyroid hormone (PTH), 25-hydroxyvitamin D [25(OH)D], creatinine, estimated glomerular filtration rate (eGFR), BTMs) and densitometric data (DXA-derived parameters, femoral neck BMD-adjusted Fracture Risk Assessment Tool (FRAX) scores) and transplant-related characteristics were collected for all participants.

Postmenopausal status was defined as the absence of menstruation for at least 12 consecutive months without physiological or pathological causes. Smoking status was recorded at the time of study assessment, and previous fragility fractures were identified from the medical records. Renal function was assessed using eGFR, calculated according to the Chronic Kidney Disease Epidemiology Collaboration (CKD-EPI) 2021 equation.

Additional transplant-related variables, such as transplant vintage, previous kidney transplantation, history and duration of hemodialysis or peritoneal dialysis, previous parathyroidectomy, maintenance immunosuppressive therapy, cumulative glucocorticoid exposure, and vitamin D analogue therapy (alfacalcidol), were collected in the KTRs. Maintenance immunosuppressive regimens comprised corticosteroids, tacrolimus, cyclosporine, mycophenolate mofetil, azathioprine, and sirolimus. Transplant vintage was defined as the time elapsed between kidney transplantation and study assessment.

### 2.4. BTM Determination

Blood samples were collected in the morning after an overnight fast into serum separator tubes containing clot activator gel. After clot formation, serum was separated by centrifugation at 4500 rpm for 5 min, aliquoted into 0.5 mL tubes, and stored at −80 °C until analysis.

BTMs were quantified using commercially available enzyme-linked immunosorbent assay (ELISA) kits according to the manufacturers’ instructions. BALP was measured using the Human ALPL/Bone Alkaline Phosphatase ELISA Kit (HUFI02249; Assay Genie Ltd., Dublin, Ireland), P1NP using the Human P1NP ELISA Kit (HUES01452; Assay Genie Ltd., Dublin, Ireland), β-CTX using the Human Beta-CTx ELISA Kit (HUES02082; Assay Genie Ltd., Dublin, Ireland), and TRAP5b using the Human Tartrate-Resistant Acid Phosphatase 5b ELISA Kit (HUFI02989; Assay Genie Ltd., Dublin, Ireland).

Serum samples were analyzed undiluted for BALP and β-CTX, diluted 1:10 for TRAP5b, and diluted 1:100 for P1NP. Optical density was measured at 450 nm using a SPECTROstar Nano microplate reader (BMG Labtech, Ortenberg, Germany), and analyte concentrations were calculated using four-parameter logistic regression standard curves. The analytical detection limits were 3.125 ng/mL for BALP, 15.63 pg/mL for P1NP, <125 pg/mL for β-CTX, and 0.313 mIU/mL for TRAP5b. The intra-assay/inter-assay coefficients of variation were 8%/10% for BALP and TRAP5b, 4.23%/4.48% for P1NP, and 4.33%/6.55% for β-CTX.

### 2.5. Biochemical Assessment

Routine biochemical analyses included serum calcium, albumin, phosphorus, magnesium, creatinine, urea, PTH, and 25(OH)D. Calcium, phosphorus, magnesium, creatinine, and urea were measured using standardized colorimetric methods on the Cobas 6000 platform (Roche Diagnostics). Serum PTH and 25(OH)D concentrations were determined by electrochemiluminescence immunoassay (ECLIA; Roche Diagnostics). Albumin-corrected serum calcium concentrations were calculated using serum albumin levels according to the formula: Corrected Calcium = (0.8 * (Normal Albumin − Patient’s Albumin)) + Serum Calcium [19].

### 2.6. DXA Assessment

BMD was assessed by DXA using a Hologic Delphi A densitometer (Hologic Inc., Bedford, MA, USA, 2001). Measurements were obtained at the lumbar spine (L1–L4), femoral neck, total hip, and one-third distal radius. For each skeletal site, BMD (g/cm^2^), T-scores and Z-scores were recorded. All DXA examinations were performed by the same trained operator using the same densitometer. Daily calibration and quality control procedures were carried out according to the manufacturer’s recommendations.

In postmenopausal women and men older than 50 years, osteoporosis was defined as a T-score ≤ −2.5 and osteopenia as a T-score between −1.0 and −2.4 according to WHO criteria. In premenopausal women and men younger than 50 years, a Z-score ≤ −2.0 was considered indicative of low bone mass for age. Finally, BMD and Z-scores (which account for age and sex) were included in the analyses. FRAX estimates, including FRAX major osteoporotic fracture risk (FRAX MOF) and FRAX hip fracture (FRAX HF), were also recorded.

### 2.7. Statistical Analysis

All statistical analyses were performed using IBM SPSS Statistics for Windows, Version 26.0 (IBM Corp., Armonk, NY, USA). Descriptive statistics were used to summarize the study population. Continuous variables were expressed as mean ± standard deviation (SD), whereas categorical variables were presented as frequencies and percentages.

Data distribution was assessed using the Shapiro–Wilk test together with skewness and kurtosis. As several continuous variables showed non-normal distributions, non-parametric methods were preferentially applied. Comparisons between KTRs and the reference group were performed using the Mann–Whitney U test for continuous variables and Pearson’s Chi-square test or Fisher’s exact test, as appropriate, for categorical variables. Mineral metabolism, bone turnover and DXA-derived parameters were compared between groups after adjustment for age, sex, BMI and postmenopausal status using ANCOVA. Adjusted between-group differences (Δ), 95% confidence intervals (CIs), and adjusted *p*-values were reported. Model assumptions were assessed by inspection of residuals. Because several biomarkers, particularly BALP, PTH, P1NP, and β-CTX, were right-skewed, adjusted models were repeated after natural log-transformation as sensitivity analyses.

Additional sensitivity analyses were performed because the reference group consisted of non-CKD individuals with DXA-confirmed osteopenia or osteoporosis. Adjusted comparisons were repeated after restricting the analysis to participants with age- and sex-appropriate low bone mass, defined as T-score ≤ −1.0 at any site in postmenopausal women and men > 50 years, or Z-score ≤ −2.0 at any site in premenopausal women and men ≤ 50 years. Covariate overlap was also assessed using propensity scores based on age, sex, BMI, and postmenopausal status. Analyses were repeated after excluding participants with non-overlapping propensity-score values, and an exploratory matched analysis was performed as an additional robustness check.

Z-scores and FRAX-derived fracture probabilities were reported descriptively and were not included in adjusted models, because Z-scores already account for age and sex, while FRAX estimates incorporate age, sex, and BMI by construction.

Correlations between BTMs, DXA-derived skeletal parameters, and calcium–phosphate metabolism parameters were assessed using Spearman’s rho. Differences in BTM levels across densitometric diagnostic categories were evaluated using the Kruskal–Wallis test. Given the number of correlation tests performed, these analyses were considered exploratory.

Receiver operating characteristic (ROC) analysis assessed each bone turnover marker’s ability to separate KTRs from non-CKD individuals with low bone mass. The binary outcome was study-group status, defined as KTR versus non-CKD reference group. Performance was expressed as the area under the curve (AUC) with 95% CI. All tests were two-tailed; *p* < 0.05 was considered significant.

## 3. Results

A total of 106 participants were included in the final analysis, comprising 50 KTRs and 56 non-CKD controls. The baseline demographic, clinical and transplant-related characteristics of the study population are summarized in Table 1. The KTRs represented a long-standing transplant cohort (mean time since transplantation 8.3 ± 6.3 years), predominantly maintained on glucocorticoids (92.0%), tacrolimus (82.0%) and mycophenolate mofetil (96.0%), with a substantial cumulative glucocorticoid exposure (mean prednisone 18.2 ± 10.7 g) and a high prevalence of CKD-MBD (84.0%; Table 1).

The KTR cohort included 23 women (46.0%) and 27 men (54.0%), whereas the control group comprised 45 women (80.4%) and 11 men (19.6%). The two groups also differed substantially in age (48.3 ± 10.5 vs. 57.7 ± 5.2 years, *p* < 0.001), BMI (27.4 ± 4.9 vs. 29.7 ± 5.1 kg/m^2^, *p* = 0.03) and proportion of postmenopausal women (24.0% vs. 80.4%, *p* < 0.001). Because these imbalances could confound the between-group comparisons of mineral metabolism, bone turnover and skeletal outcomes, all subsequent analyses were additionally adjusted for age, sex, BMI and postmenopausal status (Table 2). Z-scores and FRAX-derived fracture probabilities, however, were reported descriptively only and were not entered into adjusted models, as both already incorporate age and sex (and, for FRAX, BMI) by construction (Table 1).

When expressed as Z-scores, which compare each participant with an age- and sex-matched reference population, KTRs showed consistently lower values than controls across all measured sites, reaching significance at the total hip (−0.32 ± 0.86 vs. 0.15 ± 0.90, *p* = 0.008), femoral neck (−0.47 ± 0.97 vs. −0.12 ± 0.84, *p* = 0.048) and lumbar spine (−0.70 ± 1.17 vs. −0.41 ± 1.02, *p* = 0.038), with a similar non-significant trend at the forearm (−0.82 ± 1.35 vs. 0.07 ± 0.93, *p* = 0.191). FRAX-derived 10-year fracture probabilities were numerically higher in KTRs for both major osteoporotic fracture (3.44 ± 2.13% vs. 3.02 ± 1.51%) and hip fracture (0.85 ± 1.01% vs. 0.60 ± 1.26%), although neither difference reached statistical significance (*p* = 0.254 and *p* = 0.272, respectively, Table 1). However, no participant in either group reported a history of previous fragility fracture. Twenty-seven KTRs were receiving active vitamin D therapy (alfacalcidol, Table 1). None of the reference group subjects was undergoing treatment with active vitamin D.

After adjustment for age, sex, BMI and postmenopausal status, the elevation in PTH in KTRs remained significant (adjusted Δ = 45.7 pg/mL, 95% CI 22.2 to 69.2; *p* < 0.001), as did the reductions in eGFR (Δ = −42.6 mL/min/1.73 m^2^, 95% CI −50.9 to −34.3; *p* < 0.001) and magnesium (Δ = −0.30 mg/dL, 95% CI −0.41 to −0.19; *p* < 0.001). The lower albumin in KTRs persisted as a modest but significant difference (Δ = −0.20 g/dL; *p* = 0.033), whereas the unadjusted difference in phosphate was no longer significant after adjustment (Δ = −0.20 mg/dL, 95% CI −0.45 to 0.05; *p* = 0.123). Corrected calcium and 25(OH)D did not differ significantly between groups in the adjusted models (*p* = 0.263 and *p* = 0.077, respectively).

The markedly higher BALP concentrations in KTRs persisted after adjustment (adjusted Δ = 129.96 ng/mL, 95% CI 64.80 to 195.13; *p* < 0.001). In contrast, the lower P1NP observed in the unadjusted analysis was attenuated and no longer statistically significant once baseline characteristics were accounted for (Δ = −773.6 pg/mL; *p* = 0.602). The bone resorption markers β-CTX and TRAP5b showed no significant between-group differences in either the unadjusted or the adjusted analyses (adjusted *p* = 0.501 and *p* = 0.899, respectively).

Among BMD measurements, none of the values at the total hip, femoral neck, lumbar spine or forearm differed significantly between groups after adjustment for age, sex, BMI and postmenopausal status. In particular, the higher unadjusted lumbar spine BMD in KTRs was attenuated and no longer significant once baseline characteristics were taken into account (adjusted Δ = 0.048 g/cm^2^, 95% CI −0.004 to 0.100; *p* = 0.070), and no significant adjusted differences were observed at the other sites.

A low-bone-mass restricted sensitivity analysis was performed because the reference group consisted of non-CKD individuals with clinically documented osteopenia or osteoporosis. All 56 non-CKD reference subjects were retained and 31 of 50 KTRs met the age- and sex-appropriate low-bone-mass criteria. Results are depicted in Table 3. BALP remained significantly higher in KTRs after adjustment for age, sex, BMI, and postmenopausal status (adjusted Δ = 140.26 ng/mL, 95% CI 66.88 to 213.64; *p* < 0.001). Similar results were observed after excluding participants with non-overlapping propensity-score values (adjusted Δ = 133.48 ng/mL, 95% CI 69.58 to 197.37; *p* < 0.001). Across sensitivity analyses, the adjusted differences in PTH, eGFR, magnesium, albumin, and BALP were generally consistent with the main analysis, whereas P1NP, β-CTX, and TRAP5b did not reach statistical significance after adjustment.

Finally, because several biomarkers were right-skewed, adjusted models were repeated after natural log-transformation. BALP remained significantly higher in KTRs across the full sample, the low-bone-mass restricted analysis and the propensity-score overlap analysis, whereas P1NP, β-CTX and TRAP5b did not reach statistical significance (Supplementary Table S1).

Correlation analyses between circulating BTMs and DXA-derived skeletal parameters in KTRs are presented in Table 4. No significant associations were observed between BALP, P1NP, β-CTX, or TRAP5b and DXA-derived Z-scores at the total hip, femoral neck, lumbar spine, or forearm (all *p* > 0.05; Table 4). Similarly, no significant correlations were identified between BTM and BMD at any skeletal site or FRAX-derived estimates of major osteoporotic fracture (FRAX MOF) and hip fracture (FRAX HF), with the exception of a weak negative correlation between P1NP concentrations and forearm BMD (*ρ* = −0.285, *p* = 0.045, Table 4).

The relationships between BTM and mineral metabolism parameters and eGFR in the KTRs are depicted in Table 5. β-CTX showed a positive correlation with phosphate (r = 0.287, *p* = 0.043). No other correlations between bone turnover markers and corrected calcium, phosphate, PTH or eGFR reached statistical significance (Table 5). No additional significant correlations were found between BTMs and age, alfacalcidol treatment or transplant-related variables, such as cumulative prednisone exposure, time since transplantation, and current immunosuppressive regimen.

**Figure 2:**
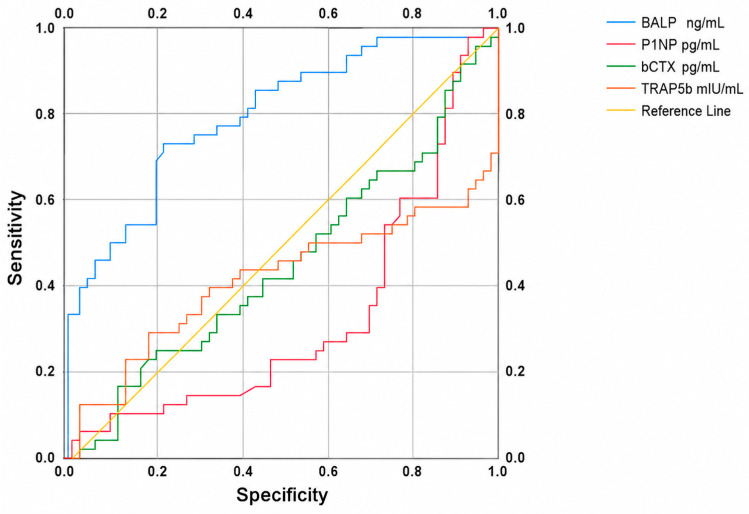
Receiver operating characteristic (ROC) curves of bone turnover biomarkers: BALP, bone-specific alkaline phosphatase; P1NP, procollagen type I N-terminal propeptide; β-CTX, C-terminal telopeptide of type I collagen; TRAP5b, tartrate-resistant acid phosphatase 5b.

Exploratory ROC analysis was performed to assess study-group status, defined as KTR versus selected non-CKD low-bone-mass reference group. BALP showed the strongest association with KTR status, with an AUC of 0.808 (95% CI 0.726–0.891; *p* < 0.001; Figure 2). P1NP showed more modest separation between groups, reflecting its lower unadjusted concentrations in KTRs (AUC = 0.682, 95% CI 0.577–0.788; *p* < 0.001), whereas β-CTX (AUC = 0.551, *p* = 0.374) and TRAP5b (AUC = 0.555, *p* = 0.364) showed no meaningful separation (Figure 2).

## 4. Discussion

Bone loss is most pronounced during the first post-transplant year, yet skeletal abnormalities frequently persist long after graft stabilization, and KTRs continue to experience an increased fracture risk (prevalence 7–20%; annual incidence 3–4%) [1,20,21]. Management remains challenging: whereas the KDIGO CKD-MBD guideline addresses the first 12 months after transplantation, it explicitly states that insufficient data exist to guide management beyond this period [5], highlighting the need for reliable non-invasive markers for long-term skeletal monitoring.

BALP reflects osteoblastic activity during the mineralization phase of bone formation and, unlike several other BTMs, is minimally influenced by renal clearance, circadian variation, or food intake. Consequently, BALP has been proposed as one of the most reliable biochemical markers for evaluating skeletal remodeling in CKD-associated osteoporosis and has also been associated with fracture risk in advanced CKD [10,13,14,21,22,23,24,25].

ROC-based discrimination of bone turnover status using BALP and related markers has previously been reported in CKD and transplant populations using bone histomorphometry as the reference standard [26]. Our analysis addresses a related but distinct question—identifying a long-standing, stable KTR population (mean 8.3 years post-transplant) from a clinically relevant non-transplant reference group. BALP demonstrated the strongest association with KTR status in our cohort, and its between-group difference was similar after adjustment for age, sex, BMI and postmenopausal status. However, this does not establish BALP as a diagnostic marker of post-transplant bone disease or as a tool for clinical decision-making. In contrast, the separation ability of P1NP should be interpreted with caution: because ROC analysis does not account for confounders, it partly reflected baseline demographic differences, consistent with the loss of a significant between-group difference after adjustment.

Our findings were consistent with previous studies describing persistent alterations in bone formation after kidney transplantation. Lloret et al. [27] demonstrated a progressive increase in BALP during the first post-transplant year, with recovery of P1NP and stable TRAP5b, suggesting gradual restoration of osteoblastic activity following the initial suppressive effects of high-dose corticosteroid therapy. Xie et al. [28] observed a rapid decline in P1NP immediately after transplantation, whereas β-CTX decreased more gradually alongside improving graft function and declining PTH levels, highlighting the distinct responses of bone formation and resorption markers during the early post-transplant period.

Histomorphometric evidence further supported the biological relevance of BALP: Ferreira et al. [29] reported that BALP correlated with mineralization defects and increased non-mineralized bone, despite overall improvement in mineral metabolism, and Keronen et al. [30] identified total alkaline phosphatase as the strongest biochemical predictor of high-turnover bone disease in KTRs.

Our results also aligned with reports of limited agreement between biochemical, densitometric, and histomorphometric assessments. Heimgartner et al. [31] demonstrated only modest associations between formation markers and subsequent BMD changes, whereas resorption markers had no predictive value; Keronen et al. [16] found no significant correlations between BTMs, DXA, and histomorphometry; and Yavuz et al. [32] reported vertebral fractures despite normal BMD, suggesting that degenerative changes and extra-osseous calcifications may artificially raise lumbar spine BMD. In our cohort, BTMs showed no consistent associations with DXA-derived Z-scores, BMD values, or FRAX-derived fracture probabilities. The isolated weak negative correlation between P1NP and forearm BMD should therefore be interpreted cautiously; given its borderline statistical significance and the number of correlation tests performed, this finding was considered exploratory. The lack of consistent associations between BTMs and densitometric measures may reflect, at least in part, the distinct biological dimensions captured by these assessments, but may also be influenced by the modest sample size, heterogeneity of the study population, and biological and analytical variability inherent to both BTM and DXA measurements. Temporal dissociation may also contribute: BTMs reflect dynamic whole-skeleton remodeling and respond rapidly to changes in osteoblast and osteoclast activity, whereas measurable BMD changes occur more gradually [26,33,34,35].

In the study by Ziolkowski et al. [36], BTMs were related to mineral metabolism, with positive associations between calcium and both β-CTX and BALP up to 24 months post-transplantation. In contrast, in our long-standing recipients (mean 8.3 years), only β-CTX correlated with serum phosphate, with no associations with calcium, PTH or 25(OH)D—likely because mineral metabolism stabilizes over time, unlike the metabolically active early period they captured. This residual β-CTX–phosphate coupling may be encountered because phosphate is less tightly regulated in KTRs, in whom hypophosphatemia is common [37]. The apparent loss of coupling between bone turnover and mineral metabolism over time represents, to our knowledge, a less-characterized feature in long-standing KTRs and suggests that BTM–mineral metabolism relationships described early after transplantation may no longer persist into the stable, long-term post-transplant period. Notably, BTMs did not correlate with cumulative glucocorticoid exposure, time since transplantation, or the current immunosuppressive regimen, suggesting that circulating bone turnover reflects ongoing remodeling rather than the historical burden of immunosuppression—though not excluding an effect of glucocorticoids in the early post-transplant period, when their impact is greatest, as reported by Ziolkowski et al. [36].

Restoration of kidney function does not fully normalize skeletal remodeling, and abnormalities may persist despite stable graft function. Schreiber et al. [38] demonstrated a decline in P1NP and β-CTX during the first six months, Sun et al. [3] reported persistent mineral abnormalities and elevated BTMs one year after transplantation in a subset of recipients, and Ferreira et al. [29] described a progressive transition from high- to low-turnover bone disease during the first year. Remodeling was also highly heterogeneous: Jørgensen et al. [39] reported marked inter-individual variability, reflecting differences in pre-transplant CKD-MBD severity, graft function, immunosuppressive exposure, hyperparathyroidism and vitamin D status. Consistent with this, Ziolkowski et al. [36] recently found that KTRs showed lower lean mass, cortical thickness and BMD at baseline, with most deficits stabilizing beyond six months except for progressive cortical thinning, which was associated with higher β-CTX, BALP and PTH, underscoring persistent resorptive activity. Fassio et al. [40] further demonstrated progressive BMD loss in untreated KTRs over four years, whereas antiresorptive therapy preserved BMD—illustrating why integrated biochemical and densitometric assessment has been proposed for long-term follow-up.

Although BTMs cannot replace DXA or bone histomorphometry, they reflect whole-skeleton remodeling activity, can be measured repeatedly during routine follow-up and may provide information not captured by PTH [9,12,13,23,25,41]. The recent 2025 European consensus on BTMs in CKD-associated osteoporosis recommends BALP and TRAP5b as reference markers of bone formation and resorption, precisely because, unlike total P1NP and β-CTX, they are not renally cleared, and emphasizes that serial measurements are likely more informative than single-time-point assessments in the post-transplant setting [10]. Our finding that BALP showed the strongest association with KTR status, whereas the difference in total P1NP did not persist after adjustment, aligns with these recommendations. Nonetheless, prospective studies incorporating serial BTM measurements, DXA, and clinically relevant outcomes are needed to determine whether BALP has prognostic or monitoring value and whether it may inform treatment decisions in long-term KTRs.

Several limitations should be considered. First, the reference group comprised non-CKD individuals with DXA-confirmed osteopenia or osteoporosis rather than metabolically healthy controls; the resulting differences in age and sex were expected and reflect a deliberate choice to compare KTRs with a clinically relevant population with established skeletal involvement. Second, the cross-sectional, single-center design precluded causal inference and limits generalizability, so prospective studies with serial BTM and DXA measurements are needed. Third, bone biopsy, which remains the gold standard for the diagnosis and classification of renal osteodystrophy, was not performed; therefore, we could not directly assess bone turnover, mineralization, or volume at the tissue level, and the BTM findings cannot be interpreted as a definitive diagnosis of the underlying bone histomorphometric phenotype. Fourth, the modest sample size may have limited power to detect weaker BTM–DXA associations. Finally, BTM interpretation is influenced by biological and analytical variability (age, sex, menopausal status, circadian rhythm, fasting, and assay methodology, particularly for β-CTX and P1NP); although samples were collected under standardized fasting morning conditions, assay differences should be considered when comparing absolute concentrations across studies. Despite these limitations, this study simultaneously evaluated multiple BTMs with DXA-derived assessment in a well-characterized cohort of KTRs and a clinically relevant reference population.

## 5. Conclusions

In this exploratory cross-sectional study, long-standing KTRs showed a distinct biochemical and densitometric profile compared with the selected non-CKD low-bone-mass reference group. Despite being younger, KTRs had lower DXA-derived Z-scores at the total hip, femoral neck and lumbar spine, suggesting a bone mass deficit relative to age- and sex-matched reference values. Among the four BTMs evaluated, BALP was the most informative, remaining significantly elevated after adjustment for age, sex, BMI and postmenopausal status and showing the strongest separation between the two selected groups in exploratory ROC analysis. In these long-standing recipients, BTMs were largely uncoupled from mineral metabolism, the only significant association being a positive β-CTX–phosphate correlation. Larger longitudinal studies including clinically relevant outcomes, serial DXA measurements and, where feasible, bone histology are required to clarify the potential role of BTMs in the skeletal assessment of long-standing KTRs.

## Figures and Tables

**Table 1:** Baseline demographic, clinical, densitometric and transplant-related characteristics of the study population.

Variable	Controls (*n* = 56)	KTRs (*n* = 50)	*p*-Value
**Baseline characteristics of the study population**			
Age (years)	57.7 ± 5.2	48.3 ± 10.5	**<0.001**
BMI (kg/m^2^)	29.7 ± 5.1	27.4 ± 4.9	0.03
Female sex, *n* (%)	45 (80.4)	23 (46.0)	**<0.001**
Male sex, *n* (%)	11 (19.6)	27 (54.0)	**<0.001**
Smoking, *n* (%)	7 (12.5)	4 (8.0)	0.51
Previous fracture, *n* (%)	0 (0.0)	0 (0.0)	—
Postmenopausal women, *n* (%)	45 (80.4)	12 (24.0)	**<0.001**
Total hip Z-score	0.15 ± 0.90	−0.32 ± 0.86	**0.008**
Femoral neck Z-score	−0.12 ± 0.84	−0.47 ± 0.97	**0.048**
Lumbar spine Z-score	−0.41 ± 1.02	−0.70 ± 1.17	**0.038**
Forearm Z-score	0.07 ± 0.93	−0.82 ± 1.35	0.191
FRAX MOF (%)	3.02 ± 1.51	3.44 ± 2.13	0.254
FRAX HF (%)	0.60 ± 1.26	0.85 ± 1.01	0.272
**Transplant-related clinical characteristics**			
Prednisone treatment, *n* (%)	—	46 (92.0)	—
Prednisone, cumulative dose (g)	—	18.2 ± 10.7	—
Tacrolimus treatment, *n* (%)	—	41 (82.0)	—
MMF treatment, *n* (%)	—	48 (96.0)	—
Alfacalcidol treatment, *n* (%)	—	27 (54.0)	—
Cyclosporine treatment, *n* (%)	—	7 (14.0)	—
Time since transplantation (years)	—	8.3 ± 6.3	—
Previous transplantation, *n* (%)	—	3 (6.0)	—
Previous hemodialysis, *n* (%)	—	29 (58.0)	—
Hemodialysis duration (years)	—	2.15 ± 2.96	—
Previous peritoneal dialysis, *n* (%)	—	7 (14.0)	—
Peritoneal dialysis duration (years)	—	0.56 ± 1.86	—
Parathyroidectomy (PTX), *n* (%)	—	1 (2.0)	—
CKD-MBD diagnosis, *n* (%)	—	42 (84.0)	—

Values are mean ± SD or *n* (%). Significant correlations (*p* < 0.05) are shown in bold. BMI, body mass index; CKD-MBD, chronic kidney disease–mineral and bone disorder; MMF, mycophenolate mofetil; PTX, parathyroidectomy; FRAX MOF, Fracture Risk Assessment Tool major osteoporotic fracture probability; FRAX HF, Fracture Risk Assessment Tool hip fracture probability.

**Table 2:** Main unadjusted and adjusted (for age, sex, BMI and postmenopausal status) comparison of mineral metabolism, bone turnover and BMD between KTRs and non-CKD reference group.

Variable	Reference Group (*n* = 56)	KTRs (*n* = 50)	Unadjusted *p*	Adjusted Effect, Δ (95% CI)	Adjusted *p*
**Mineral metabolism parameters**					
Corrected calcium (mg/dL)	9.05 ± 0.42	9.07 ± 0.56	0.837	0.16 (−0.12; 0.45)	0.263
Phosphorus (mg/dL)	3.29 ± 0.40	2.91 ± 0.61	**<0.001**	−0.20 (−0.45; 0.05)	0.123
PTH (pg/mL)	51.9 ± 17.3	93.3 ± 72.0	**<0.001**	45.7 (22.2; 69.2)	**<0.001**
25(OH)D (ng/mL)	27.9 ± 10.5	25.3 ± 9.4	0.180	−4.5 (−9.5; 0.5)	0.077
eGFR (mL/min/1.73 m^2^)	97.0 ± 9.5	58.1 ± 20.8	**<0.001**	−42.6 (−50.9; −34.3)	**<0.001**
Magnesium (mg/dL)	1.97 ± 0.15	1.64 ± 0.22	**<0.001**	−0.30 (−0.41; −0.19)	**<0.001**
Albumin (g/dL)	4.34 ± 0.44	4.27 ± 0.39	0.420	−0.20 (−0.39; −0.02)	0.033
**Bone turnover markers**					
BALP (ng/mL)	53.96 ± 57.43	233.13 ± 263.71	**<0.001**	129.96 (64.80; 195.13)	**<0.001**
P1NP (pg/mL)	7206.6 ± 4252.7	5531.1 ± 4508.0	**<0.001**	−773.6 (−3685.1; 2137.8)	0.602
β-CTX (pg/mL)	3379.4 ± 2105.6	3152.2 ± 1951.7	0.371	−320.0 (−1253.3; 613.2)	0.501
TRAP5b (mIU/mL)	1.84 ± 0.33	1.80 ± 0.71	0.327	−0.02 (−0.30; 0.26)	0.899
**BMD**					
Total hip BMD (g/cm^2^)	0.876 ± 0.109	0.896 ± 0.131	0.912	−0.012 (−0.063; 0.040)	0.650
Femoral neck BMD (g/cm^2^)	0.723 ± 0.095	0.748 ± 0.123	0.401	0.008 (−0.050; 0.066)	0.799
Lumbar spine BMD (g/cm^2^)	0.888 ± 0.092	0.934 ± 0.128	**0.038**	0.048 (−0.004; 0.100)	0.070
Forearm BMD (g/cm^2^)	0.651 ± 0.087	0.674 ± 0.088	0.121	−0.023 (−0.063; 0.017)	0.261

Values are mean ± SD. Significant correlations (*p* < 0.05) are shown in bold. KTRs, kidney transplant recipients; CKD, chronic kidney disease; PTH, parathyroid hormone; 25(OH)D, 25-hydroxyvitamin D; eGFR, estimated glomerular filtration rate; BALP, bone-specific alkaline phosphatase; P1NP, procollagen type I N-terminal propeptide; β-CTX, C-terminal telopeptide of type I collagen; TRAP5b, tartrate-resistant acid phosphatase 5b; BMD, bone mineral density; CI, confidence interval; Δ, adjusted KTR–control difference.

**Table 3:** Sensitivity analyses for adjusted between-group differences in mineral metabolism parameters and bone turnover markers.

Outcome	Main Adjusted Analysis, Δ (95% CI), *n* = 56/50	Low-Bone-Mass Restricted Analysis, Δ (95% CI), *n* = 56/31	Propensity-Score Overlap Analysis, Δ (95% CI), *n* = 49/34
Corrected calcium (mg/dL)	0.16 (−0.12; 0.45), *p* = 0.263	0.21 (−0.11; 0.52), *p* = 0.200	0.18 (−0.12; 0.48), *p* = 0.236
Phosphate (mg/dL)	−0.20 (−0.45; 0.05), *p* = 0.123	−0.17 (−0.43; 0.09), *p* = 0.197	−0.22 (−0.48; 0.04), *p* = 0.099
PTH (pg/mL)	**45.69 (22.15; 69.22), *p* < 0.001**	**48.23 (20.94; 75.52), *p* < 0.001**	**44.82 (19.78; 69.85), *p* < 0.001**
25(OH)D (ng/mL)	−4.51 (−9.50; 0.49), *p* = 0.077	**−5.56 (−10.71; −0.42), *p* = 0.034**	−4.55 (−9.67; 0.57), *p* = 0.082
eGFR (mL/min/1.73 m^2^)	**−42.62 (−50.90; −34.34), *p* < 0.001**	**−41.95 (−50.65; −33.25), *p* < 0.001**	**−41.92 (−50.05; −33.79), *p* < 0.001**
Magnesium (mg/dL)	**−0.30 (−0.41; −0.19), *p* < 0.001**	**−0.28 (−0.39; −0.17), *p* < 0.001**	**−0.29 (−0.40; −0.17), *p* < 0.001**
Albumin (g/dL)	**−0.20 (−0.39; −0.02), *p* = 0.033**	**−0.21 (−0.41; −0.02), *p* = 0.029**	**−0.22 (−0.39; −0.04), *p* = 0.016**
BALP (ng/mL)	**129.96 (64.80; 195.13), *p* < 0.001**	**140.26 (66.88; 213.64), *p* < 0.001**	**133.48 (69.58; 197.37), *p* < 0.001**
P1NP (pg/mL)	−773.63 (−3685.01; 2137.74), *p* = 0.602	−763.69 (−3907.61; 2380.24), *p* = 0.634	−499.39 (−3348.98; 2350.21), *p* = 0.731
β-CTX (pg/mL)	−320.05 (−1253.32; 613.22), *p* = 0.501	−232.98 (−1175.83; 709.86), *p* = 0.628	−231.66 (−1198.97; 735.66), *p* = 0.639
TRAP5b (mIU/mL)	−0.02 (−0.30; 0.26), *p* = 0.899	0.01 (−0.30; 0.31), *p* = 0.961	−0.04 (−0.33; 0.24), *p* = 0.769

Values are adjusted KTR–control differences, Δ (95% CI), with adjusted *p*-values. Positive Δ values indicate higher adjusted values in KTRs compared with controls, whereas negative Δ values indicate lower adjusted values in KTRs. All models were adjusted for age, sex, BMI, and postmenopausal status. The low-bone-mass restricted analysis included all 56 reference group subjects, who had clinically documented osteopenia or osteoporosis, and the 31 KTRs who met age- and sex-appropriate low-bone-mass criteria. Low bone mass was defined as T-score ≤ −1.0 at any site in postmenopausal women and men > 50 years, or Z-score ≤ −2.0 at any site in premenopausal women and men ≤ 50 years. The propensity-score overlap analysis included only participants with overlapping propensity-score values based on age, sex, BMI, and postmenopausal status. Significant correlations (*p* < 0.05) are shown in bold. PTH, parathyroid hormone; 25(OH)D, 25-hydroxyvitamin D; eGFR, estimated glomerular filtration rate; BALP, bone-specific alkaline phosphatase; P1NP, procollagen type I N-terminal propeptide; β-CTX, C-terminal telopeptide of type I collagen.

**Table 4:** Associations between bone turnover biomarkers and DXA-derived skeletal parameters in KTRs.

Marker	Total Hip BMD	Total Hip Z-Score	Femoral Neck BMD	Femoral Neck Z-Score	Lumbar Spine BMD	Lumbar Spine Z-Score	Forearm BMD	Forearm Z-Score	FRAX MOF	FRAX HF
BALP	−0.094 (0.516)	−0.034 (0.814)	−0.063 (0.665)	−0.051 (0.723)	−0.068 (0.639)	−0.056 (0.699)	−0.001 (0.992)	0.110 (0.447)	−0.001 (0.994)	0.040 (0.784)
P1NP	−0.235 (0.100)	−0.166 (0.249)	−0.109 (0.452)	−0.047 (0.745)	−0.236 (0.099)	−0.098 (0.500)	−0.285 **(0.045)**	−0.103 (0.477)	0.007 (0.963)	0.148 (0.316)
β-CTX	−0.057 (0.696)	−0.022 (0.882)	0.037 (0.801)	−0.014 (0.923)	0.026 (0.857)	0.082 (0.572)	−0.032 (0.826)	0.231 (0.106)	−0.086 (0.552)	0.077 (0.593)
TRAP5b	0.053 (0.717)	0.080 (0.579)	−0.007 (0.962)	0.016 (0.910)	0.144 (0.319)	0.212 (0.140)	−0.086 (0.552)	−0.144 (0.317)	0.078 (0.590)	0.075 (0.605)

Data are presented as correlation coefficient (ρ) with the corresponding *p*-value in parentheses. Significant correlations (*p* < 0.05) are shown in bold. BMD, bone mineral density; FRAX MOF, Fracture Risk Assessment Tool major osteoporotic fracture probability; FRAX HF, Fracture Risk Assessment Tool hip fracture probability; BALP, bone-specific alkaline phosphatase; P1NP, procollagen type I N-terminal propeptide; β-CTX, C-terminal telopeptide of type I collagen; TRAP5b, tartrate-resistant acid phosphatase 5b.

**Table 5:** Correlations between BTM and mineral metabolism parameters in KTRs.

Bone Turnover Marker	Parameter	KTRs (*n* = 50)
BALP	25(OH)D	0.036 (0.802)
	Corrected calcium	−0.035 (0.811)
	Phosphate	0.186 (0.197)
	PTH	0.251 (0.079)
	eGFR	−0.049 (0.736)
β-CTX	25(OH)D	0.123 (0.395)
	Corrected calcium	0.146 (0.313)
	Phosphate	**0.287 (0.043)**
	PTH	−0.051 (0.726)
	eGFR	−0.042 (0.772)
P1NP	25(OH)D	0.031 (0.828)
	Corrected calcium	−0.013 (0.931)
	Phosphate	0.052 (0.719)
	PTH	0.186 (0.195)
	eGFR	−0.168 (0.245)
TRAP5b	25(OH)D	0.030 (0.834)
	Corrected calcium	−0.256 (0.073)
	Phosphate	0.033 (0.821)
	PTH	0.036 (0.803)
	eGFR	0.017 (0.907)

Data are presented as correlation coefficient (ρ) with the corresponding *p*-value in parentheses. Significant correlations (*p* < 0.05) are shown in bold. BALP, bone-specific alkaline phosphatase; β-CTX, C-terminal telopeptide of type I collagen; P1NP, procollagen type I N-terminal propeptide; TRAP5b, tartrate-resistant acid phosphatase 5b; 25(OH)D, 25-hydroxyvitamin D; PTH, parathyroid hormone; eGFR, estimated glomerular filtration rate; KTRs, kidney transplant recipients.

## Data Availability

The original contributions presented in this study are included in the article/supplementary material. Further inquiries can be directed to the corresponding author.

## Supplementary Materials

The following supporting information can be downloaded at: https://www.mdpi.com/article/10.3390/jcm15155974/s1, Table S1. Log-transformed sensitivity analyses for skewed biomarkers.

## Abbreviations

25(OH)D	25-Hydroxyvitamin D
AUC	Area Under the Receiver Operating Characteristic Curve
BALP	Bone-Specific Alkaline Phosphatase
BMD	Bone Mineral Density
BMI	Body Mass Index
BTMs	Bone Turnover Markers
CI	Confidence Interval
CKD	Chronic Kidney Disease
CKD-EPI	Chronic Kidney Disease Epidemiology Collaboration
CKD-MBD	Chronic Kidney Disease–Mineral and Bone Disorder
DXA	Dual-Energy X-Ray Absorptiometry
ECLIA	Electrochemiluminescence Immunoassay
ELISA	Enzyme-Linked Immunosorbent Assay
eGFR	Estimated Glomerular Filtration Rate
FRAX	Fracture Risk Assessment Tool
FRAX HF	Fracture Risk Assessment Tool Hip Fracture Probability
FRAX MOF	Fracture Risk Assessment Tool Major Osteoporotic Fracture Probability
KDIGO	Kidney Disease: Improving Global Outcomes
KTRs	Kidney Transplant Recipients
P1NP	Procollagen Type I N-Terminal Propeptide
PTH	Parathyroid Hormone
ROC	Receiver Operating Characteristic
SD	Standard Deviation
TRAP5b	Tartrate-Resistant Acid Phosphatase 5b
WHO	World Health Organization
β-CTX	Beta-C-Terminal Telopeptide of Type I Collagen
