# Supplementary material for: Bone Turnover Markers, DXA and Mineral Metabolism in Long-Term Kidney Transplant Recipients: A Cross-Sectional Comparative Study
Source: J Clin Med. 2026 Jul 31;15(15):5974. doi: 10.3390/jcm15155974 (PMC13466847; doi:10.3390/jcm15155974)
Supplement: Supplementary file 1 [file jcm-15-05974-s001.zip › jcm-4458056-supplementary.pdf]

Supplementary Table S1. Log-transformed sensitivity analyses for skewed biomarkers

Outcome	Full sample<br>Geometric ratio KTR/control	Low-bone-mass restricted analysis<br>Geometric ratio KTR/control	Propensity-score overlap analysis<br>Geometric ratio KTR/control
**PTH**	1.74 (1.38; 2.20), p<0.001	1.78 (1.38; 2.29), p<0.001	1.74 (1.37; 2.21), p<0.001
**BALP**	**4.93 (2.72; 8.94), p<0.001**	**5.02 (2.75; 9.16), p<0.001**	**4.86 (2.66; 8.88), p<0.001**
**P1NP**	0.81 (0.56; 1.18), p=0.273	0.82 (0.55; 1.22), p=0.332	0.84 (0.60; 1.19), p=0.327
**β-CTX**	0.93 (0.71; 1.21), p=0.583	0.95 (0.73; 1.24), p=0.709	0.95 (0.72; 1.25), p=0.706
**TRAP5b**	0.94 (0.80; 1.11), p=0.480	0.95 (0.80; 1.13), p=0.586	0.93 (0.79; 1.10), p=0.384

Note. Values are adjusted geometric mean ratios for KTRs versus controls, with 95% confidence intervals and p-values. Models were adjusted for age, sex, BMI, and postmenopausal status. The low-bone-mass restricted analysis included all controls and KTRs with age- and sex-appropriate low bone mass. The propensity-score overlap analysis excluded participants with non-overlapping propensity-score values based on age, sex, BMI, and postmenopausal status. KTRs, kidney transplant recipients; PTH, parathyroid hormone; BALP, bone-specific alkaline phosphatase; P1NP, procollagen type I N-terminal propeptide; β-CTX, C-terminal telopeptide of type I collagen; TRAP5b, tartrate-resistant acid phosphatase 5b; BMI, body mass index.
